# Correlation of Oxidative Stress Markers and Nuclear Abnormalities with Clinical Parameters in Individuals with Periodontitis

**DOI:** 10.3390/dj14010050

**Published:** 2026-01-12

**Authors:** Saulo Oswaldo Sánchez-Rivera, Yveth Marlene Ortiz-Garcia, Blanca Patricia Lazalde-Ramos, Cristina Hermila Martínez-Bugarín, Guillermo Moisés Zúñiga-González, Susana Vanessa Sánchez-De-La-Rosa, Belinda Claudia Gómez-Meda, Vianeth Martínez-Rodríguez, Cristian Gabriel Guerrero-Bernal, Gabriela Morales-Velazquez, Ana Lourdes Zamora-Perez

**Affiliations:** 1 Instituto de Investigación en Odontología, Centro Universitario de Ciencias de la Salud, Universidad de Guadalajara, Guadalajara 44340, Mexico; ozswaldosrivera@gmail.com (S.O.S.-R.); yveth.ortiz@academicos.udg.mx (Y.M.O.-G.); cristybugarin21@gmail.com (C.H.M.-B.); sanvan0937@gmail.com (S.V.S.-D.-L.-R.); gmoralesv63@gmail.com (G.M.-V.); 2 Programa de Doctorado en Genética Humana, Centro Universitario de Ciencias de la Salud, Universidad de Guadalajara, Guadalajara 44340, Mexico; 3 Programa de Maestría en Ciencias y Tecnología Química, Unidad Académica de Ciencias Químicas, Universidad Autónoma de Zacatecas, Zacatecas 98160, Mexico; blancalazalde@gmail.com; 4 Laboratorio de Mutagénesis, Centro de Investigación Biomédica de Occidente, Instituto Mexicano del Seguro Social, Guadalajara 44340, Mexico; mutagenesis95@gmail.com; 5 Departamento de Biología Molecular y Genómica, Instituto de Genética Humana “Enrique Corona Rivera”, Centro Universitario de Ciencias de la Salud, Universidad de Guadalajara, Guadalajara 44340, Mexico; beligomezmeda@gmail.com; 6 Programa de Especialidad en Periodoncia, Centro Universitario de Ciencias de la Salud, Universidad de Guadalajara, Guadalajara 44340, Mexico; vianethperio@gmail.com (V.M.-R.); cristianggbernal@gmail.com (C.G.G.-B.)

**Keywords:** DNA damage, lipid peroxidation, Periodontitis, oxidative stress, saliva

## Abstract

**Background**: Chronic periodontitis (CP) is a prevalent inflammatory disease worldwide, characterized by the destruction of periodontal tissue due to an immune response triggered by periodontopathogenic bacteria and the prolonged release of reactive oxygen species (ROS). Excess ROS leads to tissue damage through mechanisms such as lipid peroxidation and DNA damage. The aim of this study was to evaluate oxidative and genotoxic damage by quantifying 8-hydroxy-2-deoxiguanosine (8-OHdG), malondialdehyde (MDA), and nuclear abnormalities (NAs) in individuals with CP. **Methods**: The participants were divided into a CP group (n = 30) and a control group without CP (n = 30). Saliva was collected to quantify 8-OHdG (via ELISA) and MDA (via spectrophotometry). Buccal mucosa samples were collected to assess NAs. Periodontal parameters, probing depth (PD), clinical attachment level (CAL), plaque index (PI), and bleeding on probing (BOP), were recorded. **Results**: The levels of 8-OHdG and MDA were significantly higher in the CP group. NAs were also significantly increased. Positive correlations were observed between 8-OHdG, MDA levels and NAs with clinical parameters. **Conclusions**: The elevated levels of 8-OHdG, MDA and NAs reflect oxidative and genotoxic damage correlated with CP severity. These biomarkers could complement diagnosis, monitor progression, and assess treatment efficacy. Their elevation may also indicate increased systemic disease risk.

## 1. Introduction

Periodontitis is one of the most prevalent chronic inflammatory diseases worldwide, affecting 20–50% of the global population and representing a significant public health burden because of its association with tooth loss, masticatory dysfunction, and systemic conditions such as cardiovascular disease and diabetes [1,2]. In Mexico, the Epidemiological Surveillance System for Oral Pathologies (SIVEPAB 2019) reported that 60% of adults have some degree of periodontitis, with the prevalence increasing with age, whereas 9% to 10% suffer from the severe form of the disease [3,4,5]. Conventional diagnostic methods, such as probing depth (PD), the presence of bleeding on probing (BOP), the plaque index (PI) and the clinical attachment level (CAL) [2,6], often detect the disease in advanced stages, highlighting the need for biomarkers that enable early diagnosis and accurate monitoring of disease progression.

Oxidative stress (OS) plays a critical role in the pathophysiology of periodontitis. An imbalance between reactive oxygen species (ROS) and antioxidant defenses leads to damage to biomolecules such as lipids, proteins and DNA [7,8,9]. This imbalance is caused by an increase in ROS production or reduced antioxidant mechanisms [10,11,12]. In periodontitis, OS released by polymorphonuclear cells in response to periodontopathogenic bacteria contributes to tissue destruction and chronic inflammation [13,14,15]. ROS-induced lipid peroxidation compromises the integrity of cell membranes, leading to direct cellular damage and impaired function [8]. At the DNA level, OS causes single or double-strand breaks and nitrogenous base modifications, disrupting cellular homeostasis and promoting apoptosis or necrosis [8,16]. Consequently, the sustained generation of ROS drives extensive cellular destruction, which further exacerbates and perpetuates the chronic inflammation and tissue degradation characteristic of periodontitis [8,14].

OS is also linked to chronic inflammatory diseases, such as atherosclerosis, cancer, Alzheimer’s disease and diabetes mellitus [14,15]. The key biomarkers for oxidative damage include 8-hydroxy-2-deoxiguanosine (8-OHdG) and malondialdehyde (MDA). 8-OHdG, a DNA damage marker, results from hydroxyl radical interactions with guanine, which leads to the formation of adducts and malformations in nitrogenous bases, reflecting the loss of cellular homeostasis [7,8,17]. MDA, a lipid peroxidation end product, disrupts cell membranes and damages DNA and proteins [18,19]. It is quantified using colorimetric techniques such as the thiobarbituric acid reaction, allowing its quantification via spectrophotometry [20].

The assay of nuclear abnormalities (NAs) in oral mucosal cells is a valuable tool for assessing genotoxic and cytotoxic damage linked to OS. NAs, including micronuclei (MN), binucleated cells (BN), nuclear buds (NBs), karyolysis (KL), condensed chromatin (CC), karyorrhexis (KR), and pyknosis (PYC), serve as markers of DNA damage and cell death [21,22]. These alterations are associated with chronic inflammatory processes such as periodontitis and reflect the impact of OS on periodontal tissues [23].

Saliva, a noninvasive biological fluid, contains biomarkers such as 8-OHdG and MDA, which are useful for evaluating OS and tissue damage in periodontal diseases [24]. Similarly, the analysis of NAs in buccal mucosa cells provides a minimally invasive method to monitor genotoxic and cytotoxic damage [23]. Previous studies have independently shown an increase in NAs and 8-OHdG levels in patients with periodontal disease [23], confirming their individual relevance. However, despite the recognized value of these markers, a comprehensive analysis that simultaneously correlates the levels of oxidative damage (8-OHdG, MDA) and genotoxic-cytotoxic effects (NAs) with the standard clinical parameters of periodontitis is lacking. It remains unclear how this specific combination of biomarkers reflects the clinical severity of the disease in an integrated manner. Therefore, this study aims to correlate the levels of 8-OHdG and MDA and the frequency of NAs with the clinical parameters in individuals with chronic periodontitis.

The alternative hypothesis of this in vivo study was that the levels of 8-hydroxy-2-deoxyguanosine, malondialdehyde, and the number of nuclear abnormalities correlate with clinical periodontal parameters in individuals with chronic periodontitis, whereas the null hypothesis was that the levels of 8-hydroxy-2-deoxyguanosine, malondialdehyde, and the number of nuclear abnormalities do not correlate with clinical periodontal parameters in individuals with chronic periodontitis

## 2. Materials and Methods

### 2.1. Study Population

There was a cross-sectional study conducted in individuals diagnosed with chronic periodontitis (CP) at the periodontics specialty clinic of the CUCS of the University of Guadalajara. The sample size was determined by applying the formula for comparing means in analytical cross-sectional studies. An attrition rate of 20% per group (representing 5 patients) was anticipated. According to the inclusion criteria (Table 1), the study groups were divided into the following groups: Group 1 (without CP): This group included 30 participants (females, n = 20, males, n = 10, mean age, 49.96 ± 3.88) with no history of periodontal disease, no clinical signs of gingival inflammation, good oral or dental health, and no evidence of clinical attachment loss, bleeding or radiographic evidence of bone loss. The gingival tissue of this group was defined as clinically healthy when the mean sulcus depth was <3 mm and there was no evidence of bleeding on probing on any surface. Thirty individuals in group 2 (with CP) (women, n = 20, men, n = 10; average age, 49.75 ± 5.37 years) presented with gingival inflammation, dental plaque formation, a probing depth of >6 mm at least six sites and a clinical attachment level of >5 mm. Inflammation was considered gingiva with redness or marked redness, edema, glazing and spontaneous bleeding.

All participants had similar socioeconomic backgrounds, and those who agreed to participate signed the informed consent letter and completed a questionnaire with personal information related to smoking habits, alcohol consumption, diseases, sex, age, dietary habits, and the consumption of medications or antioxidants. No samples were excluded from the analysis.

The sample size was calculated using the formula for comparing the means of two independent groups: $\eta =2{\left[\frac{\left({Ζ}_{\alpha }+{Ζ}_{\beta }\right)\sigma }{\varepsilon }\right]}^{2}$. A confidence level of 95% (Ζα = 1.96) and a statistical power of 95% (Ζβ = 1.65) were established. Reference values for standard deviation (σ = 84.91) and mean difference (ε = 118) were obtained from the study by Arunachalam et al. 2015 [25]. The calculation indicated a minimum of 14 subjects per group; however, 30 participants were included per group to ensure statistical robustness.

### 2.2. Periodontal Assessment

The clinical parameters that were analyzed to obtain a periodontal diagnosis were probing depth (PD), which is the distance between the gingival margin and the base of the pocket; the presence of bleeding on probing (BOP), since the inflammation present in periodontal disease, whether gingivitis or CP, can cause spontaneous bleeding or bleeding caused by manipulating the area with the periodontal probe; the plaque index (PI); and the clinical attachment level (CAL), defined as the distance between the cementoenamel junction and the bottom of the gingival sulcus and/or periodontal pocket [26]. Clinical examination was performed on all existing teeth of the participants, and periodontal status was assessed by recording PI, BOP, PD and CAL. Clinical assessment was performed with a North Carolina periodontal probe (Hufriedy, Chicago, IL, USA), and the mean probing depth was calculated. Clinical parameters were obtained from six sites of each tooth present in the participants’ mouths. The results obtained for PI and BOP are expressed as a percentage of the sites and PD and CAL are expressed as the means ± standard deviations. In addition to clinical measurements, a complete periapical radiographic study was performed, and all participants completed the anamnesis to complete the diagnosis. All measurements were performed by the same periodontal specialist and recorded on the periodontal diagnosis sheet.

### 2.3. Preparation and Analysis of Samples

Oral mucosa and saliva samples were collected from all participants. 6 mL of unstimulated saliva per participant from the study groups was collected and stored at −80 °C until analysis. A single freezing process was performed. A saliva sample was obtained 30 min after the intake of food or liquids. In group 1 (the control), a baseline sample was collected. In group 2 (the CP group), only the baseline sample was collected before any periodontal intervention was used.

### 2.4. Quantification of 8-OHdG and MDA in Saliva

The concentration of 8-OHdG in the saliva samples was determined using a competitive enzyme-linked immunosorbent assay (ELISA) kit (Cayman Chemicals, Ann Arbor, MI, USA). Saliva samples were centrifuged at 10,000 rpm for 10 min, and the determination was performed according to the kit manufacturer’s protocol. The DNA damage ELISA uses an anti-8-OHdG monoclonal antibody that competes for binding to 8-OHdG present in the sample, the standard, or 8-OHdG prefixed in the wells of a 96-well immunoassay plate. Anti-8-OHdG antibodies that interacted with 8-OHdG in the sample or the standard were removed by washing, while retained antibodies or immobilized 8-OHdG were detected with a horseradish peroxidase-conjugated secondary antibody. The assay was developed with a tetramethylbenzidine substrate, and the absorbance was measured in a microplate reader at 450 nm. The intensity of the yellow color is inversely proportional to the concentration of 8-OHdG. MDA levels in saliva were measured as an index of lipid peroxidation. MDA quantification in saliva was performed using the modified method of Yagi (1998) [27]. The saliva samples were first centrifuged at 10,000 rpm for 10 min to remove debris and cells. The resulting supernatant was used for the analysis. For the quantification of MDA, 300 µL of the clarified supernatant was mixed with 2 mL of N/12 sulfuric acid (H_2_SO_4_), 0.03 mL of 10% phosphotungstic acid (H_3_PO_4_) and 1 mL of 0.6% TBA. The mixture was then placed in a water bath at 95 °C for 45 min and then cooled. In total, 1 mL of butane was added. The MDA concentration was determined via a spectrophotometer at a wavelength of 534 nm.

### 2.5. Buccal Micronucleus Cytome Assay (BMcyt)

Buccal mucosal cell samples were collected from all participants. The participants were asked to rinse their mouths with water, and a slide was used to collect the cheek mucosa sample, which was spread on another previously cleaned and coded slide. The procedure was repeated to obtain the sample in duplicate. The smears were allowed to air dry, fixed in 80% methanol for 48 h, and stained with acridine orange (Sigma Aldrich, St. Louis, MO, USA). The staining was performed by immersing the slides in 0.008 g acridine orange solution (prepared in phosphate buffer) for 10 min. Subsequently, the slides were rinsed by immersion in phosphate buffer for 10 min to remove excess dye and allowed to air dry in the dark.

All slides were scored manually by one reader, who blindly counted nuclear abnormalities (NAs), including micronucleated (MN) cells, binucleated (BN) cells, and cells with nuclear buds (NBs) as biomarkers of genome damage, as well as cells with karyolysis (KL), karyorrhexis (KR), condensed chromatin (CC), and pyknotic (PYC) cells. The criteria used for counting the NAs were those described by Thomas et al. (2009) (Table 2) [21]. NAs were assessed in 2000 cells per sample using an OLYMPUS CX31 microscope equipped with epifluorescence and oil immersion objectives (60x and 100x; OLYMPUS, Tokyo, Japan). The results are presented as the number of cells with NAs per 2000 cells.

### 2.6. Statistical Analysis

The results are expressed as the means ± standard deviations (SD), and BOP and PI are presented as percentages. All the results were tested for normality using the Kolmogorov–Smirnov test. Differences in nuclear abnormalities (NAs), and 8-OHdG and MDA levels were analyzed using the nonparametric Mann–Whitney U test for independent samples. For correlation analysis, the Spearman test was used to assess the relationships between 8-OHdG and MDA levels, the number of NAs and clinical parameters.

A *p* < 0.05 was considered statistically significant. All tests were performed using the Statistical Program for Social Sciences (SPSS v.25) program for Windows^®^ (SPSS, Inc., Chicago, IL, USA).

## 3. Results

### 3.1. General Characteristics of the Participants

The frequency of oxidative genomic damage markers (8-OHdG, MDA and NAs) was evaluated in a sample of 60 individuals who participated in our study. Group 2, the CP group (n = 30), consisted of 20 women and 10 men with an average age of 49.75 ± 5.37 years. Group 1, control group (n = 30), consisted of 20 women and 10 men, with an average age of 49.96 ± 3.88 years (Table 3). The average age of the participants was similar and there were no differences between the groups. Neither age nor sex was used as a stratification variable because they did not present significant differences between the study groups. In terms of clinical information, individuals with CP presented a greater percentage of IP and SS, as well as significantly greater PS and NIC values, than individuals without CP did (*p* = 0.001) (Table 3).

### 3.2. Determination of Oxidative Damage to DNA and Lipids

The levels of 8-OHdG were significantly greater in individuals with CP than in those without CP (*p* = 0.001), indicating increased OS in the CP group (Table 4). Similarly, salivary MDA levels were significantly greater in individuals with CP than in those without CP (*p* = 0.001), which reflects significant lipid damage (Table 4).

### 3.3. Correlation Analysis of Salivary 8-OHdG and MDA Levels with Clinical Parameters

**Figure 1:**
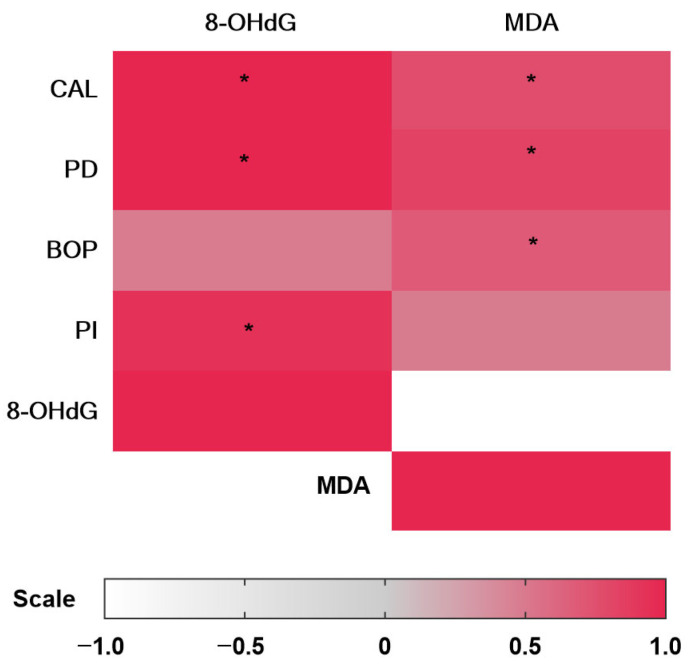
Heatmap of Spearman correlations between clinical parameters and OS markers. Statistical significance was considered when the *p* value was <0.05 (*). 8-OHdG: 8-hydroxy-2-deoxyguanosine; PI: plaque index; PD: probing depth; BOP: bleeding on probing; MDA: malondialdehyde; CAL: clinical attachment level.

A significant positive correlation was observed between salivary 8-OHdG levels and periodontal clinical parameters (CAL, PD, PI and BOP), CAL (rho = 1.000 and *p* = 0.0001), PD (rho = 1.000 and *p* = 0.0001) and PI (rho = 0.9340 and *p* = 0.0001) in the CP group (Table 5 and Figure 1). Similarly, the MDA concentration was significantly positively correlated with CAL (rho = 0.7627; *p* = 0.0001) PD (rho = 0.8206; *p* = 0.0001), BOP (rho = 0.6860; *p* = 0.0008) and PI (rho = 0.4856; *p* = 0.02) (Table 5 and Figure 1).

### 3.4. Quantification of the Number of NAs in Buccal Mucosa Cells

In the CP group, genotoxicity markers (MN, BN, NBs) were significantly greater in the MN (*p* = 0.0001), BN (*p* = 0.0001) and NB (*p* = 0.0001) groups than in the non-CP group (Table 6). In terms of the level’s cytotoxicity markers KL, KR, CC and PYC, significant increases in KL (*p* = 0.0001), KR (*p* = 0.0001) and PYC (*p* = 0.0001) were detected in the CP group compared with the non-CP group (Table 6).

### 3.5. Correlation Analysis of NAs with Clinical Parameters

**Figure 2:**
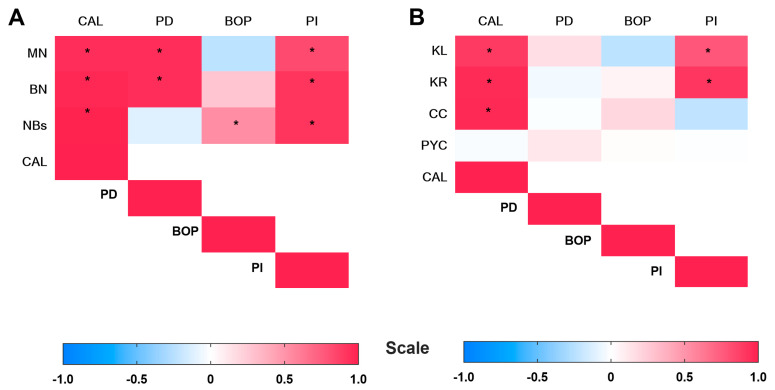
Heatmap of Spearman’s correlation coefficients between clinical parameters of ANs: (**A**) genotoxicity markers and (**B**) cytotoxicity markers. Statistical significance was set at *p* < 0.05 (*). BN: binucleated; CC: condensed chromatin; KL: karyolysis; KR: karyorrhexis; SD; standard deviation; PI: plaque index; MN: micronucleus; n: number of participants; CAL: clinical attachment level; NS: nonsignificant difference; CP: chronic periodontitis; PYC: pyknosis; NBs: nuclear buds; PD: probing depth; BOP: bleeding on probing.

A significant positive correlation was observed between the frequency of MN and the clinical parameters CAL (rho = 0.9459; *p* = 0.0001), PD (rho = 0.8458; *p* = 0.0001) and PI (rho = 0.8251; *p* = 0.0001) (Table 7 and Figure 2A). BN cells were also positively correlation with CAL (rho = 0.9727; *p* = 0.0001, PD (rho = 0.9458; *p* = 0.0001) and PI (rho = 0.9151; *p* = 0.0001), whereas NB cells were positively correlated with CAL (rho = 0.9897; *p* = 0.0001), BOP (rho = 0.5246; *p* = 0.002) and PI (rho = 0.9148; *p* = 0.0001) (Table 7 and Figure 2A). Regarding the cytotoxicity markers (KL, KR, CC, PYK), a significant positive correlation was observed between the number of KL and the clinical parameters CAL (rho = 0.8939; *p* = 0.0001) and PI (rho = 0.7717; *p* = 0.0001) (Table 7 and Figure 2B). Similarly, the KR was positively correlated with CAL (rho = 0.9559; *p* = 0.0001) and PI (rho = 0.9061; *p* = 0.0001). Moreover, CC correlated with CAL (rho = 0.9667; *p* = 0.0001) (Table 7 and Figure 2B).

## 4. Discussion

CP is an inflammatory disease characterized by increased production of ROS and an imbalance in the endogenous antioxidant system, which plays a fundamental role in tissue destruction [8,28]. Studies have demonstrated that markers of OS, such as 8-OHdG, an indicator of oxidative DNA damage, and MDA, an indicator of lipid peroxidation, are elevated in patients with PC [29,30]. The oral cavity serves as a reflection of an individual’s general health, as the characteristics of the oral mucosa can reveal changes indicative of systemic or chronic degenerative diseases, such as periodontal disease. The epithelium of the oral mucosa, which undergoes constant cell division, is an ideal tissue for biomonitoring health because of the ease of obtaining samples in a minimally invasive and painless manner. This facilitates the use of techniques such as the analysis of the NAs assay. NAs (MN, NBs, KL, KR, BC, PYC) reflect genotoxic and cytotoxic damage [23,31]. These alterations are indicative of the effect of OS on periodontal tissues and may be associated with the severity of the disease, as determined by clinical parameters [32,33,34].

This effect could be attributed to the inflammation-induced recruitment of neutrophils, which release ROS, although the respiratory burst but also generate more ROS. Additionally, neutrophils promote the citrullination of bacteria present in the CP. This effect results in the formation of immune complexes with anti-citrullinated protein antibodies (ACPAs) and increases the expression of the enzymes peptidylarginine deiminase 2 and 4 (PAD2 and PAD4), which enhances the inflammatory response and causes cytotoxic and genotoxic damage, ultimately leading to the formation of ANs [35,36].

Within the damage caused by periodontitis, one of the molecules involved is Matrix Metalloproteinases (MMPs). Since collagen is the main protein of bone, cementum and periodontal ligament, its degradation is the key event in attachment loss. In periodontitis, it has been proposed that the relationship between ROS and MMPs is bidirectional; the increase in ROS activates latent MMPs, specifically collagenase MMP-8 and gelatinase MMP-9, which are directly responsible for collagen breakdown [37]. Furthermore, MMPs (such as MMP-2) can induce increased ROS production, creating a feedback loop that perpetuates oxidative stress and tissue destruction [38].

8-OHdG is a sensitive parameter for detecting DNA damage and is the most extensively studied base damage product [8]. In this study, salivary 8-OHdG levels were significantly greater in individuals with CP than in periodontally healthy controls, supporting the concept of increased OS in CP patients. These findings align with studies such as those of Gurbuz (2024) [17] who suggested that 8-OHdG levels are significantly correlated with clinical parameters such as PD, BOP, the PI, and the CAL. This correlation is associated with the accumulation of oxidative DNA damage in tissues affected by periodontal injury [17]. Our results revealed a positive correlation between salivary 8-OHdG levels and clinical parameters such as CAL, PD and IP. Notably, the levels of CAL and PD, which are markers of periodontal inflammation and tissue destruction, were strongly positively correlated with salivary 8-OHdG levels. These findings are consistent with those of Sezer et al., who also reported a positive correlation between PD and CAL. The CAL is a clinically relevant parameter as it reflects the severity of periodontal disease and may be influenced by oxidative DNA damage in periodontal tissue. Bacterial invasion and the resulting inflammation can cause direct damage to the epithelial cells of periodontal tissues, further exacerbating OS. Similarly, Yang (2016) [39], Önder (2017) [18] and Gurbuz (2024) [17] who reported a significant correlation between 8-OHdG levels in saliva and several clinical parameters, suggest that 8-OHdG could be a useful marker of CP severity because of the increase in free radicals and the damage they generate in the cell, leading to cell death [17,18,39,40]. However, some studies, such as that of Takane et al., reported no significant correlation between salivary 8-OHdG levels and clinical parameters. These discrepancies may arise because 8-OHdG levels in gingival crevicular fluid provide more localized information about oxidative damage in specific areas of inflammation, whereas salivary 8-OHdG levels reflect overall oxidative damage in the oral cavity, including contributions from multiple sources such as oral tissues and salivary glands [8,37].

MDA has been established as an important marker of OS in individuals with CP [41]. In the present study, salivary MDA levels were significantly greater in the CP group than in the non-CP group, similar to the findings for 8-OHdG. A significant positive correlation was observed between salivary MDA levels and clinical parameters such as CAL, PD and BOP. These results agree with those published by Trivedi (2014) [42] who reported positive correlations between MDA and PI, PD, and CAL; meanwhile, Gautam (2022) [34] reported a positive correlation between CAL and 8-OHdG and MDA markers. These findings support the hypothesis that MDA reflects oxidative tissue damage and could serve as a useful marker for assessing the degree of tissue destruction in periodontal disease [34,42]. This increase in MDA levels as periodontal tissue destruction progresses is attributed to OS induced by ROS, which damages cell membrane lipids and promotes inflammation, thereby accelerating tissue destruction [43]. Salivary MDA levels exhibit good specificity for CP in systemically healthy individuals, suggesting their potential utility in assessing the local effect of periodontal disease.

NAs are indicators of genotoxic damage caused by inflammation, infection, OS, or exposure to external agents such as tobacco. They are also associated with altered regenerative processes and cellular dysplasia, which may have preneoplastic implications. In this study, the number of NAs (MN, NBs, PN, KL, and KR) in oral mucosa cells was significantly greater in individuals with CP than in those without CP. These findings indicate that NAs reflect the genotoxic and cytotoxic damage associated with chronic inflammation.

Previous studies have linked NAs to the cell destruction observed in affected periodontal tissues, suggesting that their monitoring could provide an additional indicator of tissue health in patients with CP [23,44,45]. In this study, a significant positive correlation was observed between genotoxicity markers and CAL, PD and PI. Cytotoxicity markers also correlated significantly with CAL and PI. Although there are no direct comparisons with other studies, these results suggest that the correlation between NAs and clinical parameters, particularly CAL and PD, may be due to chronic inflammation and bacterial invasion associated with CP. These processes generate OS, lipid damage and DNA damage, leading to an increase in NAs [23]. Additionally, tissue damage increases mitotic activity, which can result in errors during cell division, further contributing to the presence of NAs [46].

Finally, the correlation of these biomarkers with the clinical parameters of CP suggests that, in addition to traditional clinical methods, the evaluation of OS biomarkers (8-OHdG, MDA) and cellular damage markers (NAs) could provide valuable additional information for the diagnosis and monitoring of periodontal disease [7,8]. These markers also serve as indicators of disease progression, reflecting the degree of inflammation and cellular damage in CP [22,23]. Furthermore, they could be used to assess systemic risk, as CP is associated with systemic diseases such as diabetes and cancer. Elevated levels of 8-OHdG, MDA, and NAs may reinforce this connection [14,15]. Additionally, these biomarkers could be useful for monitoring the effectiveness of periodontal treatment, as a reduction in their posttreatment levels may indicate decreased genotoxic stress and inflammation [24]. Consistent with these findings, the statistically significant differences observed between the groups provide sufficient evidence to reject the null hypothesis, thereby supporting that chronic periodontitis is associated with increased oxidative stress and genotoxic damage.

The biological relevance of these correlations is interpreted with caution, given the cross-sectional design of the study. Rather than implying causality, the correlations suggest that greater clinical severity of periodontitis is accompanied by a higher burden of molecular damage. CAL and PD showed moderate correlations with 8-OHdG and MDA levels, indicating that loss of periodontal support and deep pocket formation are associated with increased oxidative DNA damage and lipid peroxidation. These processes may compromise fibroblast viability and cell membrane integrity, thereby favoring collagen breakdown. Moreover, the positive correlation between PI and nuclear abnormalities supports the notion that a sustained bacterial load is associated with cytotoxic effects on the oral epithelium and with cell death–related changes (such as KR and KL), which could weaken the mucosal barrier and facilitate further bacterial invasion. Taken together, these findings indicate that traditional clinical parameters are not only clinical descriptors but also reflect an underlying biological burden of oxidative and genotoxic stress within periodontal tissues. However, due to the cross-sectional nature of the study, these associations should be interpreted as indicative and hypothesis-generating, and they warrant confirmation in longitudinal studies.

Among the strengths of the study is the integrated evaluation and correlation of a panel of biomarkers reflecting different aspects of the pathogenesis of periodontitis (8-OHdG, MDA, and ANs). Unlike other investigations that often correlate these markers with four key indicators (PD, CAL, PI and BOP) for each biomarker. This not only provides robust evidence for the association but also directly links molecular damage at the DNA, lipid and cytotoxicity levels to the clinical severity of the disease.

Diet is known to be related to the modulation of oxidative stress. Diets high in glucose and saturated fats, which promote hyperglycemia, activate pro-inflammatory pathways, exacerbating the release of reactive oxygen species. This generates oxidative stress in gingival fibroblasts, hindering healing and accelerating tooth attachment loss [47]. These diets also increase markers of systemic inflammation and, consequently, oxidative stress markers such as 8-OHdG [48]. Furthermore, poor oral hygiene habits allow for biofilm accumulation, which promotes chronic inflammation and the formation of reactive oxygen species.

As has also been demonstrated, fatty degeneration within the jawbone results in the differentiation of osteoblasts into adipocytes in the jaw instead of bone. During this differentiation process, reactive oxygen species (ROS) are produced as a byproduct of the metabolism of these new fat cells, generating an oxidative stress environment that accelerates bone loss and hinders the improvement of periodontal disease [49]. However, it is worth mentioning that care was taken to ensure that our participants did not present any of these factors that could interfere with pr alter our results, through the questionnaire administered at the beginning of the study.

Regarding the study limitation, the cross-sectional design does not allow for establishing a direct causal relationship between CP and the increase in biomarkers It cannot be determined whether oxidative and genotoxic damage is a cause or a consequence of the disease. The sample size, although 30 subjects per group exceeded the minimum requirement of 13 determined by the initial power calculation to detect significant differences, this size may still limit the generalizability of the results to larger or more diverse populations. Furthermore, unlike some studies, our work did not evaluate marker levels following non-surgical periodontal treatment. Therefore, it is proposed for future research to include this assessment, as it would help demonstrate the reversibility of damage and strengthen the association found.

## 5. Conclusions

In conclusion, salivary levels of 8-OHdG, MDA and NAs provide valuable insight into the status of CP and its relationship with oxidative damage and inflammation. However, further research is needed to establish the clinical utility of these biomarkers more accurately in the context of CP.

## Figures and Tables

**Table 1:** Inclusion and exclusion criteria for selection of the study sample.

Inclusion Criteria	Exclusion Criteria
Subjects without periodontal diseases (group 1)	Individuals with chronic degenerative diseases
Subjects with periodontal diseases (group 2)	Autoimmune diseases
Both males and females	Osteoporosis
Good health and had not previously received treatment for periodontal disease	Smokers
	Pregnant women
	Individuals receiving pharmacological treatments or orthodontic

Group 1: control group, group 2: chronic periodontitis exposed group.

**Table 2:** Classification of NAs observed in buccal mucosa cells of individuals with periodontitis.

Abbreviation	Nuclear Abnormality	Morphological Features	Significance
MN	Micronuclei	Small, round nuclear fragments separate from the main nucleus	Biomarker of DNA damage and chromosomal instability
BN	Binucleated cell	Presence of two nuclei within a single cell	Reflects cytokinesis failure and potential chromosomal missegregation
NBs	Nuclear Buds	Nucleus with a budding extension containing nuclear material	Indicator of gene amplification and genomic instability
KR	Karyorrhexis	Fragmented nucleus with dense chromatin clumps	Sign of apoptotic cell death
CC	Condensed chromatin	Nucleus with dense compact chromatin	Represents early apoptosis
KL	Karyolysis	Faded or ghost-like nucleus with reduced staining intensity	Indicator of advanced cytotoxicity and cell death
PYC	Pyknosis	Highly shrunken and darkly stained nucleus	Indicates late-stage apoptosis and cell degeneration

NAs: nuclear abnormalities; MN: micronuclei, BN: binucleated cell, NBs: nuclear buds; KR: karyorrhexis; CC: condensed chromatin; KL: karyolysis; PYC: pyknosis.

**Table 3:** General characteristics and clinical parameters of the participants by group.

Study Groups			
	Without CP	With CP	*p*-Value
n	30	30	
Age (years)	49.96 ± 3.88	49.75 ± 5.37	NS
Men	10 (33.3%)	10 (33.3%)	NS
Women	20 (66.6%)	20 (66.6%)	NS
PI (%)	9.35 ± 1.74	54.84 ± 19.86	0.001 *
BOP (%)	3.90 ± 0.76	67.02 ± 16.84	0.001 *
PD (mm)	1.14 ± 0.57	3.12 ± 0.72	0.001 *
CAL (mm)	0.94 ± 0.09	4.38 ± 0.85	0.001 *

* The PI and BOP are expressed as percentage. Age, PD and CAL are expressed as the means ± SDs. Comparisons were made between groups with respect to the non-CP group. Student *t*-test was used to compare ages. The chi-square test was used to compare sex, PI and BOP and the Mann–Whitney U test was used to compare age, PD and CAL. Statistical significance was considered when *p* < 0.05 (*). n: number of individuals; PI: plaque index; BOP: bleeding on probing; PD: probing depth; CAL: clinical attachment level; NS: not significant; SD: standard deviation; PC, chronic periodontitis. %: represents the percentage obtained from the variables analyzed from the total number of participating individuals.

**Table 4:** Intergroup comparison of salivary 8-OHdG values of individuals with and without CP.

Study Groups		
	Without CP (n:30)	With CP (n:30)
8-OHdG (ng/mL)	5.75 ± 0.94 ng/mL	12.76 ± 4.55 ng/mL
*p*-value		0.001
MDA (nM/mL)	3.21 ± 0.68 ng/mL	16.75 ± 2.83 ng/mL
*p*-value		0.001

The 8-OHdG and MDA values are expressed as means ± SDs (nM/mL). Comparisons were made between groups: without CP vs. with CP, with the Mann–Whitney U test for independent samples. Statistical significance was considered when the *p* value was <0.05. 8-OHdG: 8-hydroxy-2-deoxyguanosine; SD; standard deviation; CP: chronic periodontitis.

**Table 5:** Correlation analysis between clinical parameters and salivary levels of 8-OHdG and MDA.

Markers of Oxidative Stress				
	8-OHdG		MDA	
Clinical Parameters	rho *	*p*-Value	rho *	*p*-Value
CAL	1.000	0.0001	0.7627	0.0001
PD	1.000	0.0001	0.8206	0.0001
BOP	0.4807	NS	0.6860	0.0008
PI	0.9340	0.0001	0.4856	0.02

Spearman correlation coefficient; rho * > 1: positive correlation; rho * < 1: negative correlation. Statistical significance was considered when the *p* value was <0.05. 8-OHdG: 8-hydroxy-2-deoxyguanosine; PI: plaque index; PD: probing depth; BOP: bleeding on probing; MDA: malondialdehyde; CAL: clinical attachment level; NS: not significant.

**Table 6:** Intergroup Comparison of Nuclear Abnormalities in Buccal Mucosa Cells.

Study Groups			
	Without CP	With CP	*p*-Value
Genotoxicity markers			
MN	1.26 ± 0.63	3.36 ± 1.54	0.0001
BC	2.16 ± 0.74	3.93 ± 1.43	0.0001
NBs	4.50 ± 1.25	12.60 ± 3.45	0.0001
Cytotoxicity markers			
KL	0.10 ± 0.30	1.66 ± 1.11	0.0001
KR	1.50 ± 0.04	3.26 ± 1.16	0.0001
CC	3.20 ± 1.29	4.26 ± 1.62	NS
PYC	0.23 ± 0.43	1.13 ± 0.99	0.0001

The values of the ANs (MN, NB, PN, KL, KR, CC and PYC) are expressed as means ± SDs/2000 cells. Intergroup comparisons were made between groups without PC and group with PC via the Mann–Whitney U test for independent samples. Statistical significance was considered when the *p* value was <0.05. MN: micronuclei; BC: binucleated cells; NBs: nuclear buds; KL: karyolysis; KR: karyorrhexis; CC: condensed chromatin; PYC: pycnotic cells; NS: not significant.

**Table 7:** Correlation analysis between clinical parameters and ANs: markers of genotoxicity and cytotoxicity.

	Clinical Parameters/Individuals with CP							
NAs	CAL		PD		BOP		PI	
Genotoxicity Markers	rho ^*^	*p*-Value	rho *	*p*-Value	rho *	*p*-Value	rho *	*p*-Value
MN	0.9459	0.0001	0.9458	0.0001	−0.2337	NS	0.8251	0.0001
BN	0.9727	0.0001	0.9458	0.0001	0.2630	NS	0.9151	0.0001
NBs	0.9897	0.0001	−0.1125	NS	0.5246	0.002	0.9148	0.0001
Cytotoxicity markers								
KL	0.8939	0.0001	0.1494	NS	−0.2353	NS	0.7717	0.0001
KR	0.9559	0.0001	−0.0424	NS	0.0544	NS	0.9061	0.0001
CC	0.9667	0.0001	−0.0144	NS	0.1864	NS	−0.2235	NS
PYC	−0.0291	NS	−0.1117	NS	0.0128	NS	−0.0098	NS

Spearman correlation coefficient. Statistical significance was considered when the *p* value was <0.05. rho *: Spearman correlation coefficient; rho * <1: positive correlation; rho * >1: negative correlation. BN: binucleated; CC: condensed chromatin; KL: karyolysis; KR: karyorrhexis; PI: plaque index; MN: micronucleus; CAL: clinical attachment level; NS: nonsignificant difference; CP: chronic periodontitis; PYC: pyknosis; NBs: nuclear buds; PD: probing depth; BOP: bleeding on probing.

## Data Availability

The data supporting the findings of this study are not publicly available as they are part of an ongoing research project. Data will be made available upon reasonable request once the study is completed.

## Abbreviations

The following abbreviations are used in this manuscript:

8-OHdG	8-hydroxy-2-deoxyguanosine
BN	Binucleate cells
CC	Condensed chromatin
CAL	Clinical attachment level
CP	Chronic periodontitis
ELISA	Enzyme-linked immunosorbent assay
FR	Free radical
KL	Karyolysis
KR	Karyorrhexis
MDA	Malondialdehyde
MN	Micronucleus
n	Sample size
nm	Nanometer
NBs	Nuclear buds
NAs	Nuclear abnormalities
OS	Oxidative stress
PYC	Pyknosis
PD	Probing depth
PI	Plaque index
SD	Standard deviation
